# Molecular Mechanisms of Pathogenesis, Prevention, and Therapy of COVID-19: Summarizing the Results of 2021

**DOI:** 10.3390/ijms232214210

**Published:** 2022-11-17

**Authors:** Evgenii Gusev

**Affiliations:** Institute of Immunology and Physiology, Ural Branch of the Russian Academy of Sciences, 620049 Ekaterinburg, Russia; gusev36@mail.ru

The purpose of this special issue is to highlight the main problems of the COVID-19 epidemic and to outline some ways to solve these problems, including research into the biology of the SARS-CoV-2 virus, general pathological and particular patterns of COVID-19 pathogenesis, acute and long-term complications of COVID-19, and evaluation of high-potential general and specific prevention methods and etiological and pathogenetic therapies for COVID-19. The expected benefit of this special issue is that the scientific results presented will be useful as a theoretical basis and specific practical recommendations for counteracting the COVID-19 pandemic and reducing acute complications, lethal outcome risks and long-term consequences of COVID-19.

Although a number of vaccines against COVID-19 have already been created and are used for mass vaccination, work is ongoing to design effective, safe, technologically advanced and affordable vaccines. The key objective of vaccination is to protect against COVID-19 by stimulating the production of specific antibodies to the receptor-binding domain of the spike protein (RBD) responsible for binding to cell receptors. In this vein, there is much promise in the design of a vaccine combining a recombinant spike protein and a DNA vaccine in a self-assembling particle [1]. In addition, one of the special issue articles shows that the induction of RBD-specific antibodies stimulated the activation of mature neutrophils that responded to RBD-coated particles (used as a vaccine) without causing excessive inflammation [2]. Thus, vaccination protects against COVID-19 not only through the induction of adaptive immunity, but also the activation of innate immunity. At the same time, by no means all problems of antigen-specific prevention against COVID-19 have been solved as yet. In particular, the high mutational potential of SARS-CoV-2 and the already accumulated genetic changes spawn rational uncertainty about the effectiveness of the existing vaccines [3]. In this context, the most recent variant of SARS-CoV-2 “Omicron” has raised global concerns and alarm by virtue of a large number of mutations in the spike protein facilitating virus evasion of immune protection and reducing the efficacy of the existing vaccines [4]. There are other problems that are faced in the use of different types of vaccines, such as the possibility of antibody-dependent enhancement (ADE) of viral infection [5], decrease in the effectiveness of live attenuated vaccines in the presence of neutralizing antibodies [6]; and onset of vaccination-related complications in individual patients [7,8,9]. Moreover, according to some authors, the current policy of obligatory vaccination is scientifically questionable and is likely to do more harm to society than good [10]. Another alarming fact is the current lack of global statistics on the results of the use of various types of vaccines.

However, it cannot be denied that vaccines are apparently the best means for preventing SARS-CoV-2 infections. Nevertheless, supplementing the arsenal of anti-SARS-CoV-2 vaccines with small molecules that can help expand the range of therapeutic possibilities, especially for immunocompromised patients, is currently considered an urgent need [11]. In this context, one of the promising methods to prevent acute lung damage in COVID-19 may be the blockade of SARS-CoV-2 main receptor, angiotensin-converting enzyme 2 (ACE2), on alveolocytes [12]. Computer technologies have enabled researchers to identify new directions in COVID-19 therapy associated with the modeling of several mini-protein inhibitors of RBD [13]. Computational studies have also provided an interesting suggestion for the use of theophylline as an adjuvant in the treatment of patients with COVID-19 [14]. At the same time, it should be taken into account that in silico results are not always confirmed in in vitro systems, not to mention in vivo studies [15].

The complexity of COVID-19 pathogenesis is determined by the multiplicity and multifunctionality of protein pathogenicity factors in SARS-CoV-2, their ability to block almost all stages in the production and functions of various types of interferons, disrupt the harmony between the various links of cellular stress in immunocytes, activate lymphocyte autoimmunity and polyclonal activation, initiate the development of a cytokine storm and other manifestations of systemic inflammation, as well as the ability to bind not only the main receptor—ACE2, but also additional (independent of ACE2) and cofactor receptors on the target cells [16].

The well-known risk factors for COVID-19 and development of its severe complications are pathologies associated with chronic systemic low-grade inflammation and metabolic disorders, primarily obesity, metabolic syndrome, and type 2 diabetes mellitus [17]. Metabolic dysfunctions are often associated with aging processes and endocrine dysfunctions, including as a result of decreased testosterone levels in the blood [18]. Meanwhile, there is a less studied problem of virus-induced changes in metabolic pathways known as metabolic reprogramming. Studies on COVID-19 have found significant changes in metabolism that led to the conclusion that COVID-19 is a metabolic disease [19]. It is noteworthy that metabolic reprogramming provides great opportunities for the discovery of new biomarkers and therapeutics for the treatment of COVID-19 infection.

A separate problem is the possible impact of COVID-19 on the course of chronic socially significant diseases. In particular, immune suppression caused by the SARS-CoV-2 can cause difficulties in the diagnosis and treatment of tuberculosis. Thus, long-term lymphopenia, hyperinflammation, lung tissue damage, and imbalance of CD4^+^ T cell subpopulations associated with COVID-19 may contribute to the spread of *M. tuberculosis* infection [20].

The alarming phenomenon is what the literature refers to as long COVID, long-haul COVID-19, post-COVID syndrome, chronic COVID syndrome, or post-acute sequelae of SARS-COV-2 infection (PASC) [21]. Of particular concern are the numerous overlaps in the clinical manifestations of the chronic consequences of COVID-19 (>6 months) and myalgic encephalomyelitis/chronic fatigue syndrome (ME/CFS), which is a socially significant problem of the modern world [21]. At the same time, the pathogenesis of long COVID is diverse and far from being clear throughout, including the effect on the nervous, vascular and other systems not only of SARS-CoV-2 itself, but also abnormal reactions of the organism to viral invasion [16].

It is obvious that the special issue in question was only marginally able to reflect the growing problems of COVID-19. Therefore, a new issue entitled “Molecular Mechanisms of Pathogenesis, Prevention, and Therapy of COVID-19: Summarizing the Results of 2022” has been initiated as a continuation of this topic. We hope that the new special issue will provide science-based answers to the current challenges of COVID-19.
